# Treatments and the Perspectives of Developing a Vaccine for Chagas Disease

**DOI:** 10.3390/vaccines12080870

**Published:** 2024-08-01

**Authors:** Priscila Silva Grijó Farani, Kathryn Marie Jones, Cristina Poveda

**Affiliations:** 1 Department of Biological Sciences, Border Biomedical Research Center, University of Texas at El Paso, El Paso, TX 79902, USA; 2 Department of Pediatrics, Division of Tropical Medicine, Baylor College of Medicine, Houston, TX 77030, USA; 3 Texas Children’s Hospital Center for Vaccine Development, Houston, TX 77030, USA; 4 Department of Molecular Virology and Microbiology, Baylor College of Medicine, Houston, TX 77030, USA

**Keywords:** Chagas, vaccine, new treatments

## Abstract

Chagas disease (CD) treatment and vaccine development are critical due to the significant health burden caused by the disease, especially in Latin America. Current treatments include benznidazole and nifurtimox, which are most effective in the acute phase of the disease but less so in the chronic phase, often with significant side effects. Here, using the available literature, we summarize the progress in vaccine development and new treatments that promise to reduce CD incidence and improve the quality of life for those at risk, particularly in endemic regions. New treatment options, such as posaconazole and fexinidazole, are being explored to improve efficacy and reduce adverse effects. Vaccine development for CD remains a high priority. The complex life stages and genetic diversity of *Trypanosoma cruzi* present challenges, but several promising vaccine candidates are under investigation. These efforts focus on stimulating a protective immune response through various innovative approaches.

## 1. Introduction

Chagas disease (CD), also known as American Trypanosomiasis, is caused by the protozoan parasite *Trypanosoma cruzi*. CD has become a major public and social health problem in Latin America and is considered a neglected tropical disease by the World Health Organization [1]. CD affects approximately 8–10 million people worldwide, with more than 100 million people exposed to the risk of infection [2]. However, these estimates could be higher due to underreporting and limited access to healthcare in some regions. 

Chagas disease was initially considered endemic to certain regions of Latin America, such as Bolivia, Argentina, Paraguay, Equator, El Salvador, and Guatemala, with the highest rate of infected individuals occurring in these regions [2,3]. However, due to increased globalization and the resulting higher migratory movements, CD has become a global health concern, including non-endemic countries such as the United States, Spain, Japan, and Australia. This is mainly due to the lack of monitoring and thorough screening of blood banks and the inexperience of health professionals in the diagnosis and management of CD, which contribute to non-vectorial routes of infection such as organ transplant and congenital transmission [4,5].

The life cycle of *T. cruzi* involves several stages and two main hosts: a triatomine insect (commonly known as the “kissing bug”) and a mammalian host, such as humans or other mammals [6]. The cycle begins when an infected triatomine bug takes a blood meal from a mammalian host, ingesting trypomastigotes present in the host’s blood. In the bug’s midgut, the trypomastigotes transform into epimastigotes, which multiply through binary fission. The epimastigotes then migrate to the hindgut, where they transform into metacyclic trypomastigotes, the infective form for mammals. Subsequently, the triatomine bug defecates during or after taking a blood meal, releasing metacyclic trypomastigotes in its feces. The parasites enter the mammalian host through mucous membranes, broken skin, or the bite wound itself. Once inside the host, the metacyclic trypomastigotes invade various cells (commonly muscle cells, macrophages, and nerve cells) and transform into intracellular amastigotes. The amastigotes multiply by binary fission within the host cells, eventually transforming back into trypomastigotes. The host cells burst, releasing trypomastigotes into the bloodstream, where they can infect new cells or be ingested by another triatomine bug, continuing the cycle [7,8].

The disease has two clinical phases: an acute phase and a chronic phase. The acute phase usually lasts 4–8 weeks and is characterized by the presence of amastigotes in cells adjacent to the subcutaneous tissue and circulating cells such as leukocytes and macrophages, allowing the parasite to spread through the lymphatic and blood system, lodging in other tissues [4]. This phase of the disease is marked by detectable parasitemia and inflammation caused by tissue parasitism [9]. It is usually asymptomatic or has non-specific symptoms that may include fever or flu-like symptoms that can disappear without treatment, compromising the diagnosis of the disease [4]. Patients not effectively treated during the acute phase of the disease may progress to the chronic phase after 2–3 months of infection, when the parasitemia is usually undetectable by the conventional diagnostic methods that are available [9]. Ten to thirty years after the acute phase, 60–70% of patients develop the indeterminate form of the disease and may not show any clinical signs of CD throughout their lifespan. On the other hand, 30–40% of patients evolve to specific chronic conditions of CD, with clinical manifestations of the disease’s cardiac, digestive, or cardiodigestive forms [10].

In Latin America, CD poses a significant economic burden regarding healthcare costs and the socioeconomic impact on affected individuals and communities [11,12]. It is a multifactorial load that includes increased health care costs due to long-term management and treatment, productivity loss since it affects primarily working-age individuals who may become disabled or die prematurely, and other factors such as the social and economic impact on the affected individuals, contributing to economic inequality perpetuation. Currently, CD is responsible for the loss of approximately 750,000 workdays due to premature deaths and USD 1.2 billion in lost productivity each year [12,13]. Globally, this disease is estimated to result in 50,000 deaths annually and between 1.18–5.85 Disability-Adjusted Life Years (DALYs) [14], implying a global economic burden of around USD 7 billion per year due to both the cost of treatment and financial losses caused by lost productivity by or morbidity of infected people [11]. 

Therefore, the importance of developing treatments and/or vaccines for CD cannot be overstated. Effective treatments can reduce long-term management needs and prevent disability or premature death among working-age individuals, thereby improving their quality of life and reducing the global burden of the disease, which currently affects millions of people worldwide. Prophylactic or therapeutic vaccines would provide a preventative measure, potentially eradicating or significantly reducing the incidence of CD in endemic and newly endemic areas. Thus, in this review, we focus on current and prospective new treatments and the perspective for either prophylactic or therapeutic vaccine candidates for CD.

## 2. Treatment

### 2.1. Current Treatment

The etiological treatment of CD mainly aims to eliminate the *T. cruzi* parasite, consequently improving patients’ clinical outcomes and interrupting the disease transmission cycle [15]. Only two drugs are available to treat the infection: Benzidazole (BZN) and Nifurtimox (NFX), which have been consistently used to treat the disease over the last 60 years [3]. BZN is currently on the WHO list of essential medications [1,16] and is part of a group of nitro heterocyclic compounds containing one or more nitro groups linked to an aromatic ring [15]. This substance is a prodrug which exerts its effect after the type I trypanosomal nitro-reductase enzyme activation present in *T. cruzi* and other protozoa, producing reactive metabolites that have a trypanocidal effect on the intra- and extra-cellular forms of the parasite [15,17]. On the other hand, NFX is a nitrofuran compound whose mechanism of action consists of producing reduced oxygen metabolites—nitroanions—after drug activation by the parasite’s nitroreductases in the presence of oxygen. This leads to the production of free radicals that block DNA synthesis and accelerate DNA and RNA degradation, vital cellular components for *T. cruzi* [12,17,18]. However, their efficacy and safety data are not ideal, as both drugs have a high rate of adverse effects [17].

Treatment is effective during the acute phase of the disease—showing a 60–85% cure rate—in addition to reducing the severity of symptoms, shortening the clinical course, and decreasing the detection of parasitemia. Unfortunately, infected people are not always correctly diagnosed or treated because their broad symptoms are often mistaken for symptoms of other more common diseases [17]. Recent data has shown that less than 1% of infected patients are diagnosed and treated correctly [12], reaffirming the need for an accurate initial diagnosis so that therapeutic interventions are carried out early [3]. Conversely, in 1983 a panel of experts recommended not treating patients in the chronic phase. This recommendation was based on limited evidence of the effectiveness of etiological treatment in this phase and the belief that the characteristic symptoms of chronic CD resulted from an exacerbated immune response unrelated to the parasite. However, there was irrefutable evidence that parasite persistence acted as a triggering factor for chronic CD pathology [15]. Since then, the efficiency of treatment in the chronic phase has been under debate, as studies have shown that treatment in the chronic phase could be associated with delayed progression of the disease, although only 15% of patients show negative seroconversion after ten years [19]. Therefore, several clinical trials have been carried out with both drugs (Table 1) to establish whether the pharmacotherapy traditionally used for treating CD is effective in negative serological and molecular tests or capable of retarding the progression of the chronic disease [17].

### 2.2. New Treatments

Although there was significant improvement in new drug discovery regarding other trypanosomatid diseases, such as African trypanosomiasis and leishmaniasis, developing new medications for CD has proven more challenging [28]. Evaluation of new treatments for CD focuses on reducing the side effects of the current treatment (BZN and Nifurtimox), reducing parasite burden, and curing or preventing the chronic phase of CD, but there have been significant challenges in finding molecules that can achieve complete parasite clearance.

Recently, Padilla et al. identified a highly effective compound of a class of benzoxaboroles, AN15368, for treating CD. The results showed a uniform infection cure in non-human primates with long-term naturally acquired infections of diverse *T. cruzi* genetic types [29]. Results indicate that AN15368 is safe, elicits no significant side effects, and is more effective than existing drugs, with clinical trials expected to start in the next few years. Since 2009, the Drugs for Neglected Diseases initiative (DNDi) has been raising efforts for massive drug screening in order to find new drug candidates for CD. However, only the benzoxarobole compound DNDI-6148 showed promising results and has progressed to phase I studies [30].

Fexinidazole was approved in 2018 for treating sleeping sickness, and it is a nitroimidazole compound that has shown promise as a potential treatment for CD as an oral treatment of acute and chronic experimental CD [31]. Ravuconazole (E1224) is an antifungal drug that has demonstrated activity against *T. cruzi* in an experimental acute murine model. In the early stages of infection, administering optimal doses of E1224-benznidazole combination treatment led to a 100% cure rate. However, this approach was unable to eradicate an already well-established disease. Nonetheless, the combination therapy showed significant benefits, as it further reduced parasitemia compared to using either drug alone [32]. New drug formulations have also been tested for CD (Table 2), mainly in the chronic stage of infection.

## 3. Vaccines

As CD is a significant public health concern in Latin America, developing a preventive/therapeutic vaccine would have an enormous economic impact in the region [14]. An effective vaccine could provide long-term protection against the *T. cruzi* parasite, preventing new infections and reducing the costs associated with diagnosis, treatment, and management of the disease, consequently reducing morbidity/mortality and the burden on public health costs [38]. For example, up to USD 1.2 million would be saved if a therapeutic vaccination strategy was applied in pregnant women to avoid the progression of CD in infected children [39]. 

However, the development of a vaccine for CD faces several challenges that need to be addressed:

- **Complex parasite life cycle:** *T. cruzi*, has a complex life cycle involving different stages and multiple forms of the parasite, and each stage may require a different type of immune response for effective control and eradication [6].
- **Limited understanding of protective immunity:** The precise immune response required to confer protection against CD is not fully understood yet [40,41]. Identifying the key immune mechanisms involved in controlling the infection and developing vaccines that elicit those specific responses is essential to creating an effective CD vaccine.
- **Lack of predictive animal models:** Animal models used in preclinical studies may not fully recapitulate the complex dynamics of CD in humans. Therefore, it can be challenging to accurately predict the effectiveness of vaccine candidates in humans based on animal studies alone [42,43].
- **Lack of surrogate markers of protection:** To date, no established surrogate markers can reliably predict vaccine efficacy against CD [44]. The absence of such markers makes it challenging to assess the efficacy of vaccine candidates in clinical trials and may require long-term follow-up to determine their effectiveness.
- **Limited commercial interest:** CD primarily affects marginalized and economically disadvantaged populations, predominantly in Latin America. The lack of financial incentives for pharmaceutical companies has historically hindered the development of vaccines for neglected tropical diseases. Public–private partnerships and alternative funding mechanisms might be necessary to overcome this challenge [45].
- **Regulatory and manufacturing challenges:** Vaccine development involves navigating complex regulatory processes and scaling production to meet global demand [46]. Regulatory approvals, manufacturing infrastructure, and cost-effectiveness are significant challenges that must be addressed to ensure access to an affordable and widely available CD vaccine [45].

**Figure 1:**
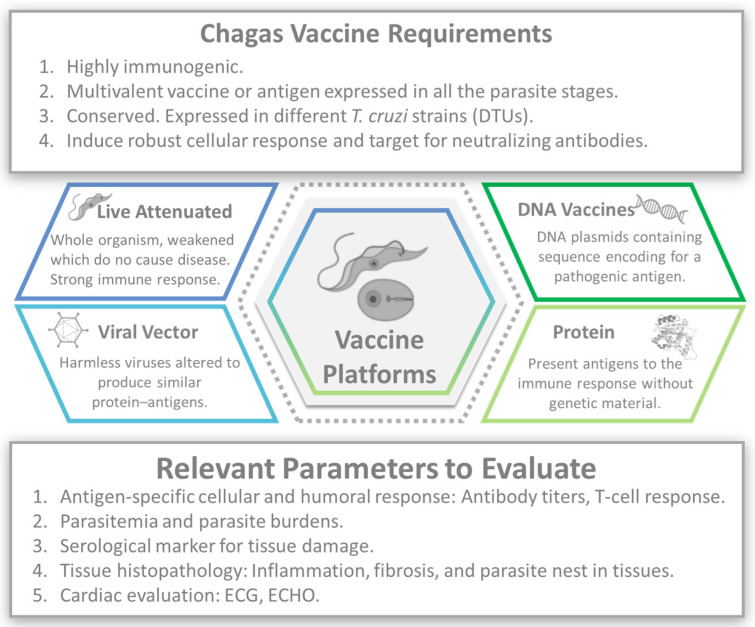
Chagas vaccine development. Image created with the assistance of BioRender.

Nevertheless, vaccines are one of the most effective tools for preventing infectious diseases [47]. Several preclinical vaccine candidates for CD have been investigated in animal models. These candidates aim to elicit an immune response against *T. cruzi*, with humoral and cell-mediated immune responses to extracellular trypomastigotes and intracellular amastigotes (Figure 1).

A wide array of vaccination strategies has been conducted to date, including, but not limited to, nucleic acids, protein subunits, attenuated vaccines, and nanoparticles, which have been assessed in mice with promising results [48,49].

### 3.1. Attenuated Vaccines

Attenuated vaccines have been instrumental in controlling and eliminating several infectious diseases, including polio, yellow fever, varicella, rotavirus, influenza, measles, mumps, and rubella. These types of vaccines induce strong and lasting immunity. Despite their effectiveness, they pose challenges, especially in immunocompromised individuals. However, these vaccines are highly effective in veterinary medicine, contributing significantly to the control and prevention of animal diseases, which make a great type of vaccines for controlling CD. Live attenuated vaccines involve modifying the parasite to reduce its virulence while maintaining its immunogenicity, which has been successfully tested in other protozoa, including *Trypanosoma brucei*, *Leishmania major*, and *Plasmodium*.

In the case of CD, the genetic manipulation of *T. cruzi* has been more challenging and only few attenuated-parasite approaches have been used as vaccines in preclinical models. Some of the vaccine candidates include an attenuated parasite with the deletion of the *lyt-1* gene and the parasite factor involved [50]. A second approach was the deletion of the surface glycoprotein 72 (*gp72),* which affects the morphology of the parasite in multiple stages of the life cycle [51]. A third approach deletes the enoyl-coenzyme A (CoA) hydratase 1 and 2 genes (ECH1^+/−^ ECH2^−/−^), two enzymes involved in the amastigote metabolism. The last approach is the deletion of the bifunctional enzyme dihydrofolate reductase-thymidylate synthase (DHFR-TS) [52]. These attenuated parasites fail to establish persistent infection, can induce strong immune responses, and protect against subsequent *T. cruzi* challenges through an increment in the CD8^+^ T-cell immune response [53]. One of the biggest challenges of attenuated vaccines, particularly in the case of attenuated parasites, is obtaining sufficient quantities of the parasite in consistent quality for use in vaccinations. Variability in parasite quality can affect the immune response elicited by the vaccine, leading to inconsistent protection. However, the use of attenuated vaccines in animals is often more acceptable and practical than in humans due to a combination of risk tolerance, economic considerations, and regulatory environments. All of these candidates could potentially decrease the number of reservoirs that infect triatomine bugs. 

### 3.2. DNA Vaccines

DNA vaccines work by introducing a small piece of the pathogen’s DNA into the body, which encodes specific proteins or antigens. These vaccines have been widely explored as a potential approach for developing a vaccine against CD, as the produced antigens stimulate an immune response, triggering the production of antibodies and activating immune cells to recognize and neutralize the pathogen. To date, multitudes of *T. cruzi* DNA vaccine studies have been conducted (Table 3) using several well-characterized *T. cruzi* virulence factors. For example, cruzipain is a significant cysteine-protease enzyme and GP82 is a surface glycoprotein of *T. cruzi* that plays a role in host cell invasion [54]. The amastigote surface protein-2 (ASP-2) and TcG1 are *T. cruzi* antigens expressed in the parasite’s metacyclic trypomastigote form, which is involved in the parasite’s entry into host cells [55]. When mice were immunized with recombinant *Trypanosoma cruzi* small surface protein 4 (rTcSSP4), they became more susceptible to trypomastigote infection, and this resulted in high mortality rates. On the other hand, when mice were immunized with a eukaryotic expression plasmid containing the TcSSP4 cDNA, the acute phase of infection was effectively controlled. The difference in outcomes between the two forms of TcSSP4 immunization highlights the importance of understanding the type of immune response generated by a vaccine candidate and how it influences protection against the target pathogen [56]. Finally, other studies have been used trans-sialidases, with the administration of CpG or the cytokine IL-15, effectively in mice to reduce mortality and chronic disease [57,58,59]. 

### 3.3. Viral Vector

Viral vectors are a type of vaccine that uses a harmless virus to deliver genetic material into the human body’s cells (Table 4). The genetic material typically encodes a specific antigen from *T. cruzi* to stimulate an immune response without causing the disease. These viral vectors have been used as gene delivery systems for recombinant vaccines and gene therapy applications, since unlike subunit vaccines they can elicit a humoral response without needing an adjuvant. Other advantages of the viral-vector vaccines are the strong and durable immune responses, versatility in design, genetic stability, and production efficiency. However, viral-vector vaccines have several challenges and potential problems, including pre-existing immunity that can neutralize the viral vector vaccine and reduce efficacy and safety concerns, including insertional and mutagenic concerns. Virus vectors expressing the *T. cruzi* antigens TSSA, TS, and ASP-2 induce specific antibody-secreting B cells and cytotoxic T cells with elevated levels of IFN-γ, resulting in reduced parasitemia and an increment in survival [60,61]. 

### 3.4. Recombinant Protein or Peptide Vaccines

Recombinant protein vaccines use purified or synthesized parasite proteins to induce an immune response, and similar to the *T. cruzi* DNA vaccines, protein vaccines have been investigated as potential candidates for generating a vaccine against CD. These include well-characterized *T. cruzi* antigens capable of priming the immune system with potent humoral and cellular immunity to confer protection [48,62]. Some of these *T. cruzi* antigens include trans-sialidase, amastigote surface proteins, or cruzipain. In addition to whole-protein vaccines, subunit vaccines are also potential candidates and typically consist of specific protein fragments or peptides derived from the parasite. These vaccines aim to focus the immune response on crucial antigenic regions of the parasite while reducing potential side effects [63]. Some preclinical studies have evaluated subunit vaccines containing peptides or recombinant protein fragments derived from *T. cruzi*, which have shown promising results in terms of immunogenicity and protection (Table 5).

Promising results have been reported from the surface proteins, Tc24 and trypomastigote small surface sntigen-1 (TSA-1), using aluminum phosphate as an adjuvant, and were therapeutic (BALBc, c57BL/6 and ICR) and/or preventive (BALBc) in their treatment outcome. BALBc and c57BL/6 mice with acute infection, and ICR mice with chronic infection showed and induction of balanced humoral and Th1/Th2/Th17 cellular responses have a protective efficacy against *T. cruzi* [69,70].

### 3.5. Glycoconjugates

The glycocalyx of *T. cruzi* consists of abundant, complex, highly variable, and immunogenic glycosylphosphatidylinositol (GPI)-anchored glycoproteins and glycolipids [72]. These include mucins, mucin-associated surface proteins (MASPs), TS/gp85 glycoproteins, and glycoinositolphospholipids, which vary throughout the parasite’s life-cycle stages [73]. Among the glycoproteins present on trypomastigotes, the GPI-anchored mucins (tGPI-mucins) carry the immunodominant glycotope Galα1,3Galβ1,4GlcNAc (Galα3LN) and several branched α-Gal-terminating O-glycans that have not been fully characterized yet. The presence of these α-Gal glycotopes stimulates the production of high levels of CD-specific anti-α-Gal antibodies in CD patients. These antibodies play a protective role by effectively controlling the parasitemia during the disease’s acute and chronic phases [74,75] and are found in abundance among patients from endemic and non-endemic countries. These molecules aim to induce an immune response against the parasite to provide protection against the disease. These proteins have effectively protected against CD [76] and leishmaniasis [77] and could potentially serve as vaccine candidates for acute and chronic CD (Table 6).

### 3.6. Multivalent Vaccines

A multivalent vaccine combines multiple antigens from the same or different pathogens into a single vaccine formulation. This approach is often used to target multiple strains or stages of a pathogen and enhance the vaccine’s overall effectiveness [78]. For example, TcG1–TcG8 are 8 vaccine antigens phylogenetically conserved in clinically important strains of *T. cruzi* that are expressed in the infective (trypomastigote) and intracellular (amastigote) parasite stages. These vaccine candidates elicited varying levels of lytic antibody response and Th1 cytokines (IFN-γ), a property associated with immune control of *T. cruzi*. TcG1-, TcG2-, and TcG4-encoded antigens were expressed on the plasma membrane of the mammalian stages of *T. cruzi* (trypomastigote/amastigote) and elicited significant levels of anti-parasite lytic antibody responses in a micNA-prime/protein-boost subunit vaccine (TcVac2) [79].

DNA vaccines composed of ASP-1, ASP-2, and TSA-1 have been shown to provide partial protection from lethal *T. cruzi* infection, and importantly, they can significantly reduce the severity of chronic CD. Glycosylphosphatidylinositol (GPI)-anchored proteins from *T. cruzi*—ASP-1, ASP-2, and TSA-1—that are the targets of CD8^+^ cytotoxic T lymphocytes (CTLs) and that induce strong antibody responses in infected mice and humans belong to the *T. cruzi* trans-sialidase family. Trans-sialidases are enzymes that transfer sialic acid residues from host glycoconjugates to the parasite’s own surface glycoproteins, allowing the parasite to modulate host immune responses and evade the immune system [80]. However, it’s also important to note that despite controlling *T. cruzi* infection and the beneficial effects on CD severity, the vaccines using the three genes tested in the study failed to inhibit infection or eliminate parasites from infected animals completely (Table 7). Moreover, they were unable to prevent death from infection in 100% of vaccinated animals. These results indicate that while the genetic vaccines targeting these specific proteins look promising and positively impact disease progression, they are not fully effective in providing complete protection against *T. cruzi* infection or eradicating the parasite in all cases [81].

### 3.7. Heterologous Vaccines

Heterologous vaccination refers to the administration of different types of vaccines for the primary and booster doses of a vaccination schedule. In contrast, homologous vaccination involves using the same vaccine for both the initial and booster shots. Despite potentially raising development costs and complicating production, heterologous vaccination holds the promise of providing superior immunity. As a result, it becomes particularly compelling for vaccine development efforts targetting complex pathogens like parasites [83]. A heterologous strategy can optimize T-cell responses to elicit robust polyfunctional T-cell responses, especially considering that multiple boosts in the same immunization site may lead to T-cell sequestration (Table 8). This approach can effectively trigger cell-mediated immunity against parasites [84].

### 3.8. mRNA Vaccines

Developing a vaccine for CD poses several challenges due to the parasite’s life cycle complexity and the variety of immune responses required for protection. Nonetheless, researchers have made progress in exploring potential vaccine candidates and studying the immunological responses needed to combat the infection. Technological advancements in vaccine development provide immunity against infectious diseases by utilizing various cutting-edge technologies and methodologies, including mRNA vaccines [88]. The Tc24 antigen was recently evaluated in a heterologous mRNA prime/protein boost strategy, showing promising results that include an improvement in the humoral and cellular responses with robust polyfunctional cells, with the equivalent-balanced Th1/Th2/Th17 secreted-cytokine profile required for complete protection against *T. cruzi* [89]. However, efficacy data is needed to understand if the immune profile obtained is able to control the infection. Recently, a study explored the development and potential of mRNA-based vaccines targeting *T. cruzi* antigens Tc24 and ASP-2 as a therapeutic option for CD, showing promising results in reducing parasite burdens and cardiac inflammation in a chronic mouse model [88].

Several notable challenges hinder the widespread clinical application and large-scale mRNA vaccine immunization programs, particularly for the Latin American region. These obstacles include a lack of quality control measures, inconsistent purification standards, insufficient manufacturing capabilities, storage inadequacies, management supervision issues, and suboptimal biocompatibility [90,91].

## 4. Vaccine-Linked Chemotherapy

An interesting approach for treating CD is vaccine-linked chemotherapy. This approach combines the antiparasitic drug BZN to reduce parasite load with the vaccine antigen, boosting the cellular and humoral immune responses. The first studies focused on vaccine-linked chemotherapy evaluated the recombinant protein vaccine Tc24-C4 + TLR4 adjuvant and showed promising results not only in a reduction of parasite burden, but also in a reduction of cardiac inflammation and fibrosis. The scheme has been tested on acute and chronic infection models using the H1 strain of *T. cruzi.* This strategy allowed a reduction in the dose of BZN to 25 mg/kg per mouse, which will benefit the patients by minimizing the side effects of the treatment and making it more tolerable [92,93,94,95]. Testing of this strategy in a chronic model of infection showed improved cardiac structure and function and cardiac metabolism [94,95]. Similarly, the recombinant protein vaccine antigen TS + ISPA was evaluated in conjunction with the curative dose of BZN (100 mg per mouse) and showed a reduction in parasite burdens and reduced cardiac arrhythmias [96]. These studies showed that combining vaccine-linked chemotherapy has enhanced efficacy in BNZ treatment and offers a dose-sparing approach that may improve overall cardiac health and clinical outcomes.

## 5. Discussion

The development of new treatments and a vaccine for CD has faced numerous scientific and logistical challenges. Traditional treatments with benznidazole and nifurtimox are plagued by side effects and limited efficacy in chronic stages of the disease, underscoring the need for better therapeutic options [97]. As highlighted in this review, most clinical trials for CD focus on optimizing new regimens of the already extensively used medications, Benznidazole and Nifurtimox [97]. Benznidazole, a nitroimidazole derivative, has been the cornerstone of Chagas disease treatment for decades due to its efficacy in the acute and early chronic stages of the disease. Nifurtimox, another nitrofurane-based compound, similarly offers benefits in these stages [17]. However, both drugs are associated with significant side effects, such as gastrointestinal disturbances and neurological symptoms, which often lead to treatment discontinuation [26].

Recent trials have aimed to mitigate these adverse effects by experimenting with reduced dosages and shorter treatment durations. For instance, new regimens have included lower doses of Benznidazole over shorter periods, which have shown promise in maintaining efficacy while improving patient tolerance [20,21,25,26]. Additionally, combination therapies involving Benznidazole or Nifurtimox with other drugs are being explored to enhance therapeutic outcomes and reduce the parasite load more effectively [33,34,37]. The potential of nitroheterocyclic compounds like fexinidazole and its metabolites demonstrated superior efficacy in murine models compared to benznidazole [31,98]. Furthermore, CYP51 inhibitors such as posaconazole [36,99] and ravuconazole [33] have shown promising results in preclinical trials. The combination of nitroheterocyclic drugs with azoles was particularly effective, suggesting that multi-drug regimens could improve therapeutic outcomes. The application of nanomedicine, such as formulations in nanocarriers [100,101], also displayed outstanding efficacy against various *T. cruzi* strains, indicating a potential path forward for overcoming drug resistance and enhancing treatment efficacy that could potentially be applied to other molecules. Thus, these efforts are crucial for increasing the accessibility and effectiveness of Chagas disease treatment, particularly in resource-limited settings where the disease is most prevalent [102].

On the other hand, the development of both prophylactic and therapeutic vaccines has been a focal point of research to combat this disease, but despite significant efforts, an effective vaccine remains elusive due to the complex life cycle of *T. cruzi* and the varied immune responses required to control its different stages. Early attempts at inoculation of mice with attenuated strains showed only partial immunity. While many vaccination strategies have been explored, including the use of attenuated strains, cultural forms, and molecular components of *T. cruzi*, none have achieved sterile immunity [103]. Several studies have made significant strides in understanding the immunological mechanisms of *T. cruzi*. For instance, the importance of CD8^+^ T cells and IFN-γ in controlling *T. cruzi* infection and the discovery of key antigen candidates such as TS, ASP-2, Tc24, and the α-Gal epitope, which have shown promise in inducing protective immunity in murine models [103]. Additionally, the potential use of recombinant adenoviruses encoding these antigens to induce long-term protective immunity in animal models was also explored [48].

Despite these advances, translating findings from animal models to human applications remains a significant hurdle. Animal studies have limitations in predicting human responses due to biological differences between species, leading to vaccine efficacy and safety discrepancies. The predictive value of animal models is often questioned, with a growing consensus that alternative methods, such as specific in vitro models and organ-on-chip technologies, might offer better predictive capabilities [43,104].

## 6. Perspectives

Over the course of several decades, the relentless pursuit of a CD vaccine has persisted, yet we find ourselves still lacking an approved vaccine for broad-scale immunization programs, especially in regions where CD is endemic [105]. It is imperative to underscore that vaccine development is a complex process, and it may take time before an effective vaccine for CD becomes available. However, as highlighted in this review, developing a CD vaccine is a highly dynamic and vigorously pursued field of research, and several potential vaccine candidates are being continuously investigated [63].

Given the complexity of the *T. cruzi* parasite, which includes different life stages and a broad genetic diversity that might influence vaccination and treatment effectiveness, a multifaceted approach might be required to develop a successful CD vaccine. Additionally, efforts towards the advancement of vaccine development to preclinical and clinical trials are needed to move the best candidates up to the next level and closer to a regulatory agency-approved vaccine to be used in the patients effectively. This involves collaborative efforts of international organizations, governments, research institutions, and industry to accelerate this much-needed tool against CD [48].

The use of vaccines against *T. cruzi* in veterinary medicine could provide significant benefits, especially for domestic and wild animals that serve as reservoirs for the parasite. For example, rabies vaccines for domestic animals have been crucial in reducing transmission to humans through animal bites, with widespread implementation decades before human vaccines were developed [106]. This underscores the proactive role of veterinary vaccines in mitigating zoonotic disease risks and influencing subsequent human vaccine developments. Vaccinating animals can help reduce the transmission of *T. cruzi* to humans and decrease the prevalence of Chagas disease in endemic areas. By controlling the disease in animal populations, the risk of human infection can be significantly diminished, particularly in rural and peri-urban areas where human–animal interactions are frequent. 

Veterinary vaccination programs can be integrated with other control measures, such as vector control and improved housing conditions, to create a comprehensive strategy against Chagas disease. Veterinary vaccines against *T. cruzi* will likely be implemented more quickly than human vaccines, and this rapid deployment can play a crucial role in controlling the disease in animal populations, thereby reducing the transmission risk to humans. While veterinary vaccines alone may not eliminate Chagas disease, they are a critical component of an integrated approach to managing and potentially eradicating this public health threat.

Furthermore, we must emphasize the necessity of unwavering commitment to the advancement of vaccine development. Transitioning promising research findings and potential candidates from the laboratory bench to the real-world context of preclinical and clinical trials is a pivotal step in the journey to provide a safe and effective CD vaccine for patients, especially those at risk in endemic areas. This dedicated and rigorous approach ensures that we navigate the challenging terrain of vaccine development with due diligence, bringing us closer to a solution for developing a successful CD vaccine.

## Figures and Tables

**Table 1:** Clinical trials for Chagas disease using Benznidazole or Nifurtimox.

Name	Treatment Regimen	Stage of Disease	Target Patients	Country	Status
CHICO SECURE (Chagas disease in children treated with Nifurtimox with follow-up for seroconversion and cure) [20]	NFX 8–20 mg/kg/day (30 days) NFX 8–20 mg/kg/day (60 days)	Acute and chronic	Pediatric patients <18 years old	Multicentric (Argentina, Bolivia and Colombia)	Ongoing
TESEO (new therapies and biomarkers for Chagas disease) [21]	BZN 300 mg/day (60 days) BZN 150 mg/day (30 days) BZN 150 mg/day (90 days) NFX 480 mg/day (60 days) NFX 480 mg/day (30 days) NFX 240 mg/day (90 days)	Indeterminate or early cardiac compromised	Adult patients (18–50 years old)	Bolivia	Ongoing
BETTY (Short-course Benznidazole treatment to reduce *Trypanosoma cruzi* parasitic load in women of reproductive age) [22]	BZN 300 mg/day (60 days) BZN 150 mg/day (30 days)	Chronic in postpartum period	Postpartum *T. cruzi* positive women patients	Argentina	Ongoing
EQUITY [23]	BZN 300 mg/day (60 days) BZN 150 mg/day (120 days) NFX 480 mg/day (60 days) NFX 240 mg/day (120 days)	Chronic Indeterminate	Adult patients (20–65 years old)	Colombia	Ongoing
MULTIBENZ (Evaluation of different Benznidazole regimens for the treatment of chronic Chagas disease) [24,25]	BZN 300 mg/day (60 days) BZN 400 mg/day (15 days) BZN 150 mg/day (60 days)	Chronic	Adult patients (>18 years old)	Multicentric (Spain, Brazil, Argentina, Colombia)	Completed
BENEFIT (Benznidazole evaluation for interrupting trypanosomiasis) [26]	BZN 300 mg/day (80 days)	Chronic cardiomyopathy	Adult patients (18–75 years old)	Multicentric (Brazil, Argentina, Colombia, Bolivia and El Salvador)	Completed
TRAENA (Tratamiento con benznidazol en pacientes adultos con enfermedad de Chagas crónica de bajo riesgo) [27]	BZN 200 mg/day (60 days)	Chronic	Adult patients (20–55 years old)	Argentina	Completed

Completed: completed clinical trial with published results; Ongoing: incomplete clinical trials or completed clinical trials with unpublished results.

**Table 2:** Clinical trials for Chagas disease proposing the use of new medications.

Name	Medication and Regimen	Target Patients	Target Patients	Country	Status
BENDITA (Benznidazole; new doses, improved treatment, and therapeutic associations) [33]	BZN 300 mg/day (60 days) BZN 300 mg/day (30 days) BZN 300 mg/day (15 days) BZN 150 mg/day (30 days) BZN 150 mg/day (30 days) + Forsvuconazole (300 mg/day 3 days + 300 mg/week) BZN 150 mg/week (60 days) + Forsvuconazole (300 mg/day 3 days + 300 mg/week)	Chronic Indeterminate	Adult patients (18–50 years old)	Bolivia	Completed
E1224 [34]	E1224 4000 mg (8 weeks) E1224 2000 mg (8 weeks) E1224 2400 mg (4 weeks) + Placebo (4 weeks) BZN 5 mg/kg/day (60 days) Placebo (8 weeks)	Chronic Indeterminate	Adult patients (18–50 years old)	Bolivia	Completed
STOP-CHAGAS [35]	Posoconazole 800 mg/day (60 days) BZN 400 mg/day + Placebo 10 mg/day (60 days) Posoconazole 800 mg/day + BZN 400 mg/day (60 days) Placebo 10 mg/day (60 days)	Chronic Indeterminate	Adult patients (18–50 years old)	Multicentric (Argentina, Chile, Colombia Guatemala, Mexico, and Spain)	Completed
CHAGASAZOL [36]	Posaconazole 800 mg/day (60 days) Posaconazole 200 mg/day (60 days) BZN 300 mg/day (60 days)	Chronic Indeterminate	Adult patients (>18 years old)	Multicentric (Bolivia, Brazil and Paraguay)	Completed
Oral Fexinidazole Dosing Regimens for the Treatment of Adults With Chronic Indeterminate Chagas disease [31,37]	Fexinidazole 600 mg/day (10 days) Fexinidazole 1200 mg/day (3 days) + Placebo 1200 mg/day (7 days) Fexinidazole 600 mg/day (3 days) + Fexinidazole 1200 mg/day (4 days) + Placebo (3 days)	Chronic Indeterminate	Adult patients (18–60 years old)	Spain	Completed

Completed: completed clinical trial with published results; Ongoing: incomplete clinical trials or completed clinical trials with unpublished results.

**Table 3:** An overview of the DNA vaccines and their efficacy for prophylaxis of Chagas disease in mouse models. Only the studies that assessed most of the parameters listed below were selected and referenced in the table.

Vaccine Antigen	Mouse Model/Infection Route/*T. cruzi* Strain	Dose/ Immunization Route	Parasite Burden /Survival	Antibody Response	Cellular Immune Response
Cruzipain [54]	BALBc/ SC/Tulahuen	2 doses, 1 week apart/IM	Reduction/ Increase	Cruzipain-specific IgG (Sera) and secretory IgA (Fecal)	Cytolytic activity Increased CD8^+^ and levels of IFN-γ
ASP-2 or UB-ASP-2 [55]	C57BL/6-PA28 knockout (PA28α/β^−/−^) and LMP2 or LMP7 knockout (LMP2^−/−^ or LMP7^−/−^)/SC/Tulahuen	4 doses, 2-week intervals /SC	Reduction/ Increase	NR	Cytolytic activity Increased levels CD8^+^ and IFN-γ
TcSSP4 [56]	BALBc/IP/H8	2 doses, 2-week intervals/IP	Reduction/ Increase	NR	Increased levels IL-10 and IFN-γ
Transialidase [57,58]	BALBc/ SC/Tulahuen	2 doses, 1 week apart/IN	Increase	TS-specific IgG (Sera) and secretory IgA (Fecal)	Increased levels CD8^+^ and IFN-γ
TS + IL-15 [59]	BALBc/SC/Tulahuen	3 doses, 2-weeks intervals/IM	NR/Increase	No differences	Increased levels CD8^+^ and IFN-γ

SC: Subcutaneous; IN: Intravenous; IM: Intramuscular; IP: Intraperitoneal. TS: Transialidase.

**Table 4:** An overview of the viral vector vaccines and their efficacy for prophylaxis of Chagas disease in mouse models. Only the studies that assessed most of the parameters listed below were selected and referenced in the table.

Vaccine Antigen	Mouse Model/Infection Route/*T. cruzi* Strain	Dose/ Immunization Route	Parasite Burden/Survival	Antibody Response	Cellular Immune Response
Adenovirus expressing TSSA CD8^+^ epitope [60]	C57BL/6/IP/Tulahuen lethal and sublethal	2 doses 12 days apart/Prime IM and boost IM or IP	Reduction/ Increase	NR	Increased levels IFN-γ
Adenovirus expressing TS and ASP-2 [61]	BALBc and C57BL/6/ IP/Y	Multivalent 2 doses, 6- to 8-week interval/SC	Reduction/ Increase	NR	Cytolytic activity Increased levels IFN-γ

TSSA: Trypomastigote small surface antigen; TS: Transialidase; ASP-2: Amastigote surface protein-2; SC: Subcutaneous; IN: Intravenous; IM: Intramuscular; IP: Intraperitoneal; NR: Not reported.

**Table 5:** An overview of the recombinant protein vaccine platform and their efficacy against Chagas disease in mouse models. Only the studies that assessed most of the parameters listed below were selected and referenced in the table.

Vaccine Antigen	Mouse model/Infection Route/*T. cruzi* Strain	Scheme/Dose/ Immunization Route	Parasite Burden/Survival	Antibody Response	Cellular Immune Response
ASP-2 [64]	A/Sn, C3H/HeJ and C3H/HePAS/ IP/Y	Prophylactic/3 doses at 0, 2 and 4 weeks/IP	Reduction/ Increase	NR	Increased CD8^+^
rCruzipain Adjuvant: CpG ODN [65,66]	C3H/HeN (H-2K haplotype)/ NR/RA	Prophylactic/2 doses, 1 week apart/IM	Reduction/ NR	IgG	Increased levels IFN-γ, IL-2, and IL-10
rGP82 Adjuvant: CpG ODN [67]	BALBc/ conjunctival or oral/ Tulahuen	Prophylactic/2 doses, 2 weeks apart/IN	Reduction/ Increase	NR	Increased levels IFN-γ
MASPpep-KLH Adjuvant: Al(OH)_3_ [68]	C3H/HeNsd; BALBc/ IP/Y	Prophylactic/3 doses, 10–15 days/IP	Reduction/ Increase	IgG	Increased levels IL-4, IL-10, IFN-γ and IL-12
rTc24 Adjuvant: E6020-SE/MPLA/CpG [58,69,70]	BALBc/IP/H1	Prophylactic/2 doses, 2 weeks apart/IM	Reduction/ Increase	IgG2a	Increased levels IFN-γ
	BALBc/IP/H1	Therapeutic/2 doses, 4 weeks apart/SC	Reduction/ NR	NR	Increased CD8^+^, Increased levels IFN-γ
	ICR/IP/H1	Therapeutic/2 doses, 2 weeks apart/SC	Reduction/ NR	Balanced IgG1/IgG2a	Increased levels IFN-γ
TS Adjuvant: CpG ODN [41,71]	BALBc/ SC/Tulahuen	Prophylactic/2 doses, 1 week apart/IN	Reduction/ Increase	TS-specific IgG (Sera) and secretory IgA (Fecal)	Increased CD8^+^ and levels of IFN-γ

ASP-2: Amastigote surface protein 2; TS: Trans-sialidase; IP: Intraperitoneal; IN: Intranasal; IM: Intramuscular; FP: Footpad; PO: Oral gavage; NR: Not reported.

**Table 6:** An overview of the recombinant protein vaccine platform and its efficacy against Chagas disease in mouse models. Only the study that assessed most of the parameters listed below was selected and referenced in the table.

Vaccine Antigen	Mouse Model/Infection Route/*T. cruzi* Strain	Scheme/Dose/ Immunization Route	Parasite Burden/Survival	Antibody Response	Cellular Immune Response
Galα3LN-HSA [76]	C57BL/6/IP/CL Brener	Prophylactic/4 doses at 0, 1, 2 and 3 weeks/IP	Reduction/ Increase	IgG, IgG1, IgG2b, IgG3	Increased CD4^+^, Increased CD8^+^, Increased CD4^+^ CD44^+^

IP: Intraperitoneal.

**Table 7:** An overview of the multivalent vaccines and their efficacy against Chagas disease in mouse models. Only the studies that assessed most of the parameters listed below were selected and referenced in the table.

Vaccine Antigen	Vaccine Platform	Mouse Model/ Infection Route/*T. cruzi* Strain	Scheme/Dose/ Immunization Route	Parasite Burden/Survival	Antibody Response	Cellular Immune Response
TS and ASP-2 [61]	Viral vector	BALBc and C57BL/6/ NR/Y	Prophylactic/SC 2 doses, 6- to 8-week interval/SC	Reduction/ Increase	NR	Cytolytic activity Increased levels IFN-γ
TSA-1, ASP-1 and ASP-2 with IL-12 and GM-CS [80]	DNA	C57BL/6/IP/Brazil	Prophylactic/ 2 doses, 6 weeks apart/IM	Reduction/ Increase	Antibodies	Cytolytic activity
TSA-1 and Tc24 [82]	DNA	BALBc/IP/H1	Prophylactic and therapeutic/2 doses, 2 weeks apart/NR	Reduction	NR	Increase antigen specific T cells.
		BALBc and C57BL/6/IP/ H1	Therapeutic/2 doses, 2 weeks apart during acute phase/NR	Reduction	NR	NR
		ICR/IP/H1	Therapeutic/2 doses, 2 weeks apart during chronic phase/NR	NR	NR	Increase CD8^+^

ASP-2: Amastigote surface protein 2; TS: Trans-sialidase; IP: Intraperitoneal; IN: Intranasal; IM: Intramuscular; SC: Subcutaneous; PO: Oral Gavage; NR: Not reported.

**Table 8:** An overview of the heterologous schemes and their efficacy in Chagas disease in mouse models. Only the studies that assessed most of the parameters listed below were selected and referenced in the table.

Vaccine Antigen	Vaccine Platform	Animal Model/ Infection Route/*T. cruzi* Strain	Scheme/Dose/Immunization Route	Parasite Burden/Survival	Antibody Response	Cellular Immune Response
rASP2 [85]	Recombinant Protein DNA	A/Sn inbreed/ IP/Y	Prophylactic Protein/DNA/3 doses at 0, 3 and 5 weeks/IP	Reduction/ Increase	NR	Increased CD8^+^, IFN-γ
TcVac2 (TcG1, TcG2, TcG4) + IL-12 + GM-CSF [86]	DNA and recombinant protein boost + saponin	C57BL/6/IP/ Sylvio X10/4	Prophylactic/5 doses, 3 of DNA vaccine with 2 weeks interval and 2 doses of recombinant protein/IM	Reduction/ NR	Increased IgG, IgG1, and IgG2b	Increased CD8^+^, IFN-γ
DNA + adenovirus expressing TS and ASP-2 clone 9 (Prime-Boost) [87]	DNA and viral vector	C57BL/6 and A/Sn/Y/SC	Prophylactic/DNA Prime and viral vector 21 days after prime vaccination/IM	Reduction/ Increase	NR	Cytolytic activity Increased levels CD8^+^ and IFN-γ
Traspain + CDA, Nt-Cz + ASP2 + CDA [84]	DNA and protein	C3H/HeN (H-2k)/Lethal RA and chronic Clone K-68/IP	Prophylactic/4 doses, 2 doses of each antigen, dose interval of 10 days/Prime PO − boost IN	Reduction/ Increase	NR	Increased polyfunctional cells

ASP-2: Amastigote surface protein 2; TS: Trans-sialidase; CDA: STING agonist, 3′5′-c-di-AMP; IP: Intraperitoneal; IN: Intranasal; IM: Intramuscular; SC: Subcutaneous; PO: Oral Gavage; NR: Not reported.
